# High Expression of TIMELESS Predicts Poor Prognosis: A Potential Therapeutic Target for Skin Cutaneous Melanoma

**DOI:** 10.3389/fsurg.2022.917776

**Published:** 2022-05-19

**Authors:** Shixin Zhao, Shifeng Wen, Hengdeng Liu, Ziheng Zhou, Yiling Liu, Jinbao Zhong, Julin Xie

**Affiliations:** ^1^ Department of Burn Surgery, First Affiliated Hospital of Sun Yat-Sen University, Guangzhou, China; ^2^ Department of Orthopedics, Guangzhou First People’s Hospital, School of Medicine, South China University of Technology, Guangzhou, China; ^3^ Department of Dermatology, Guangzhou Institute of Dermatology, Guangzhou, China

**Keywords:** skin cutaneous melanoma, prognostic signature, overall survival, timeless, immune infiltration, bioinformatics

## Abstract

**Background:**

Skin cutaneous melanoma (SKCM) is the most lethal skin cancer with an increasing incidence worldwide. The poor prognosis of SKCM urgently requires us to discover prognostic biomarkers for accurate therapy. As a regulator of DNA replication, TIMELESS (TIM) has been found to be highly expressed in various malignancies but rarely reported in SKCM. The objective of this study was to evaluate the relationship between TIM and SKCM tumorigenesis and prognosis.

**Methods:**

We obtained RNA sequencing data from TCGA and GTEx to analyze TIM expression and differentially expressed genes (DEGs). Subsequently, GO/KEGG, GSEA, immune cell infiltration analysis, and protein-protein interaction (PPI) network were used to perform the functional enrichment analysis of TIM-related DEGs. Moreover, the receiver operating characteristic (ROC) curves, Cox regression analysis, Kaplan–Meier (K-M) analysis, and nomograms were applied to figure out the clinical significance of TIM in SKCM. In addition, we investigated the relationship between TIM promoter methylation and SKCM prognosis through the UALCAN database. Finally, the immunohistochemical (IHC) results of normal skin and SKCM were analyzed to determine expression differences.

**Results:**

TIM was significantly elevated in various malignancies, including SKCM, and high expression of TIM was associated with poor prognosis. Moreover, a total of 402 DEGs were identified between the two distinct TIM expression groups, and functional annotation showed enrichment with positive regulation of cell cycle and classic oncogenic pathways in the high TIM expression phenotype, while keratinization pathways were negatively regulated and enriched. Further analysis showed that TIM was correlated with infiltration of multiple immune cells. Finally, IHC validated the differential expression of TIM in SKCM.

**Conclusion:**

TIM might play a pivotal role in tumorigenesis of SKCM and is closely related to its prognosis.

## Introduction

Skin cutaneous melanoma (SKCM) is the most lethal skin cancer with an increasing incidence worldwide, accounting for 287,723 new cases and 60,712 deaths in 2018 (1, 2). For patients with primary SKCM, complete resection is currently considered as the first choice, providing the highest probability of cure (3). However, there are quite limited means to treat patients with metastatic SKCM. Despite the significant progress in the adjuvant therapies of SKCM, many important questions remain, such as toxicity of chemotherapy, drug resistance, and expensive cost (3). Thus, increasing incidence worldwide, poor prognosis, and limited efficacy of available therapeutic tools prompted us to undertake extensive mechanistic investigations to discover novel prognostic biomarkers and new targets for therapy that might support precision medicine.

TIM, first identified in drosophila and subsequently in mammals, is not only involved in circadian rhythms, but also in cell cycle and DNA replication . Moreover, TIM also participate in cell survival after DNA damage or replication stress by promoting TIPIN nuclear locallization and play an important role in epithelial cell morphogenesis (4). Anomalous cell cycle and molecular clockwork have been implicated in various diseases, notably tumorigenesis (5). Indeed, TIM has been demonstrated to be overexpressed in several malignancies compared to normal tissues, such as colorectal cancer, small cell lung cancer, and breast cancer (6–8). Not only that, the expression of TIM has been significantly associated with advanced tumor stages as well as poor prognosis (9). Therefore, TIM might has the potential to be used as a biomarker of cancer susceptibility, diagnosis, and prognostic outcome. Should this be the case, TIM could be a worthy therapeutic target.

In recent years, growing studies have shown that molecular factors (BRAF, MEK, PD-1) are important for biology, drug targeting, and prognosis in SKCM (10–12). To date, however, comprehensive studies of TIM in SKCM have yet to be reported. The focus of this study was to ascertain TIM expression level in SKCM and its potential prognostic significance. Moreover, the mechanisms by which TIM influences the prognosis of SKCM and its potential relationship to immune infiltration were also discussed.

## Materials and Methods

### RNA-Sequencing Data and Ethics Statement

TPM expression values were downloaded from the UCSC XENA Project (https://xenabrowser.net/datapages/), which contains the TCGA and GTEX RNA-Seq data that was processed uniformly to provide more reliable expression analysis with tumor and normal samples (13, 14). Besides, clinicopathological information and corresponding RNA-seq data of SKCM in FPKM format were downloaded from the TCGA database (https://portal.gdc.cancer.gov/). Apart from 1 patient with incomplete clinicopathological information, a total of 471 SKCM patients were included. In this study, RNA-seq data was converted from FPKM to TPM format for further analysis. Since the UCSC XENA and TCGA database is publicly available in accordance with certain guidelines, it acknowledges that all written informed consents have been obtained before collecting data.

### Differentially Expressed Genes (DEGs) in SKCM Tumors

According to the cut-off value of 50%, 471 SKCM patients were divided into low- and high-TIM expression groups. The “DESeq2” R package was used to identify DEGs between the two groups, where the log-fold change greater than 1.5 and adjusted *P*-value less than 0.05 were set as thresholds (15). The “ggplot2” R package was used to present the results in the form of volcano plots and heatmaps.

### Functional Enrichment Analysis of TIM-Associated DEGs

The identiﬁed DEGs were then processed for functional enrichment analysis. The “clusterProfiler” and “ggplot2” R packages were used to analyze and visualize the functions of GO, including cell composition (CC), molecular function (MF), and biological process (BP), as well as KEGG pathway analysis (16).

### Gene Set Enrichment Analysis (GSEA) of TIM-Associated DEGs

The “clusterProfiler” R package was utilized for the GSEA of DEGs to clarify the functional differences between the two subgroups (16). 1,000 permutations of the gene set were performed for each analysis. We selected C2: curated gene sets from MSigDB collections as reference gene sets. Clusters with adjusted *p*-value (*p*.adj) less than 0.05 and false discovery rate (FDR) less than 0.25 were identified as statistically significant (17). The “ggplot2” R package were used for GSEA visualization.

### Relationship Between TIM and Immune Cell Infiltration in SKCM Tumors

The “GSVA” R package was utilized to analyze inﬁltration enrichment of 24 common immune cells, including activated dendritic cells (aDCs); B cells; CD8 T cells; Cytotoxic cells; DCs; Eosinophils; immature DCs (iDCs); Macrophages; Mast cells; Neutrophils; NK CD56bright cells; NK CD56dim cells; NK cells; Plasmacytoid DCs (pDCs); T cells; T helper cells; T central memory (Tcm); T effector memory (Tem); T follicular helper (Tfh); T gamma delta (Tgd); Th1 cells; Th17 cells; Th2 cells; Treg (18, 19). Subsequently, the spearman analysis was used to further verify the relationship between TIM expression and immune cell infiltration.

### Association of Clinicopathological Characteristics and TIM Expression in SKCM Patients

The Wilcoxon rank sum test or Pearson’s chi-square test were used to compare the clinicopathological characteristics of the two distinct TIM groups. The overall survival (OS) was calculated from the date of diagnosis to the date of death or last follow-up. The disease specific survival (DSS) was calculated from the date of diagnosis to the date of death caused by SKCM. Univariate Cox proportional hazards regressions were utilized to identify the individual hazard ratio (HR) for the OS and DSS in SKCM patients. Subsequently, the significant variables in the univariate analysis (*p < *0.05) were further subjected to multivariate analysis. The HR of individual factors was estimated by 95% confidence interval (CI).

### Clinical Signiﬁcance of TIM Expression in SKCM

In order to test the predictive accuracy of TIM for SKCM diagnosis, the “pROC” and “ggplot2” R packages were used to analyze and visualize ROC curves. Prognostic data of SKCM patients, including OS and DSS, was derived from a published study (20). The “survival” R package was used for statistical analysis of survival data, and the “survminer” R package was applied for visualization of K-M curves. Finally, the R package “rms” and “survival” were applied for generating nomograms and calibration plots (20).

### Promoter Methylation and Genetic Alterations of TIM in SKCM

In order to determine if there is a relationship between TIM promoter methylation and tumor severity, we assessed the correlation between TIM promoter methylation status and cancer stages, nodal metastasis status in patients who enrolled in the UALCAN (http://ualcan.path.uab. edu/). Moreover, data and analysis of TIM mutation in SKCM were obtained from the cBioPortal database (http://www.cbioportal.org) and the images were also generated using the cBioPortal database. To further elucidate possible molecular mechanisms, we used STRING database (https://cn.string-db.org/) to construct protein-protein interaction (PPI) network and perform functional enrichment analysis.

### IHC Validation of TIM Expression

To validate the results obtained in the bioinformatic prediction analysis, we collected IHC results of normal skin and SKCM tumors from the human pathology proteinatlas (HPA) database. 4 normal skin tissues and 14 SKCM tumors were collected. The integrated optical density (IOD) of each image and the corresponding stained area were measured using ImageJ 1.8.0 software. The mean optical density (MOD) was obtained by calculating the ratio, and the mean MOD (hereafter referred to as OD value) of three random fields was used to represent the expression level of TIM protein in SKCM and normal skin tissues.

### Statistical Analysis

All statistical analysis and graphs were performed and visualized by R (3.6.3), and *P*-value less than 0.05 was considered as statistically significant.

## Results

### Expression Profiles of TIM in Pan-Cancers and Related Differentially Expressed Genes in SKCM

**Figure 1:**
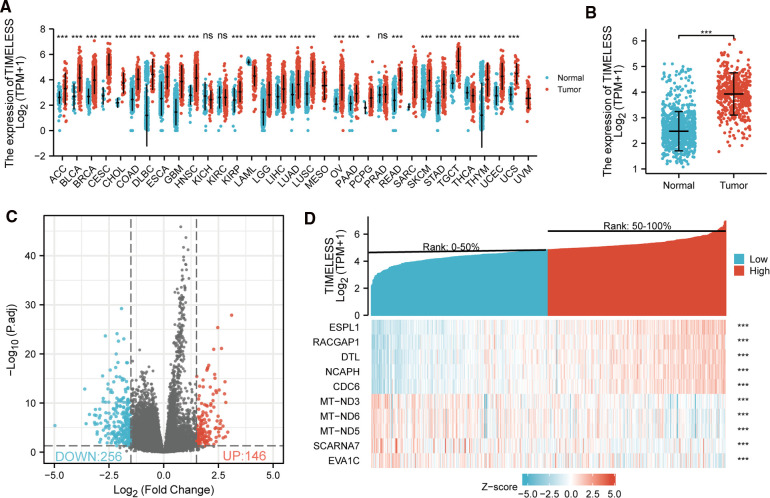
Differential mRNA expression profiles in skin cutaneous melanoma (SKCM) patients stratiﬁed by TIM levels. (**A**) The comparison of TIM expression between tumor and normal tissue in different malignancies based on TCGA database. ns, *p* ≥ 0.05; *p < 0.05; **p < 0.01; ***p < 0.001. (**B**) TIM expression was higher in SKCM tumors than normal skin tissue. Based on the median TIM level, 472 SKCM patients from TCGA-SKCM project were stratiﬁed into high- and low-TIM expression groups. Shown were expression proﬁles of mRNA in the two subgroups; and data were presented by volcano plots (**C**) and heatmaps (**D**).

Based on TCGA database, the expression of TIM mRNA in different cancers were determined. As shown in Figure 1A, among 33 cancer types, the TIM was significantly elevated in 25 cancers. More particularly, TIM expression was much higher in SKCM than in normal skin tissues (*p* < 0.001, Figure 1B). Interestingly, only two of the investigated cancer profiles was TIM expression decreased.

472 SKCM patients were stratiﬁed into two subgroups based on the median TIM expression. Next, we compared mRNA expression between the two groups. Finally, 402 mRNAs (146 upregulated and 256 downregulated, Figure 1C) were recognized as DEGs (absolute value of log_2_FC > 1.5, *p*.adj < 0.05) between the two subgroups. Representative DEGs were also illustrated by heatmap (Figure 1D).

### Functional Annotation of TIM-Associated DEGs in SKCM

**Figure 2:**
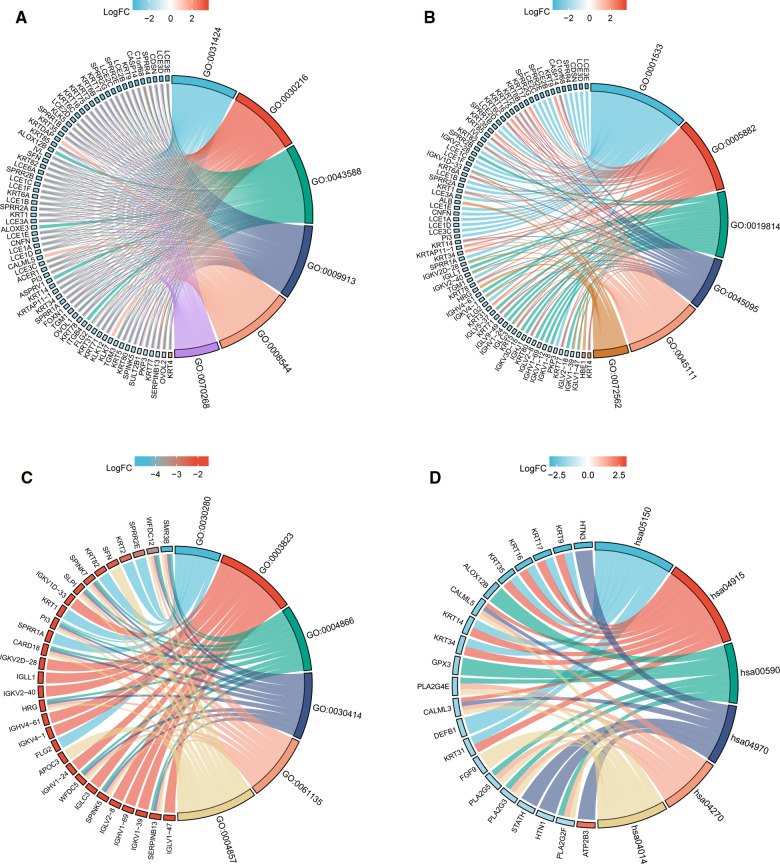
GO/KEGG enrichment analysis of TIM-related DEGs in TCGA-SKCM project. (**A**) Enriched GO terms in the “BP” category; (**B**) Enriched GO terms in the “MF” category. (**C**) Enriched GO terms in the “CC” category; (**D**) KEGG pathway annotations. The right semicircle represents different functional categories, while the left semicircle consists of individual DEGs. Different colors indicate different properties, and the size of the sections represents the number of DEGs.

In order to understand the functional implication of TIM-associated DEGs, GO and KEGG functional enrichment analysis was performed by clusterProfiler package (Supplementary Table S1 and Figure 2). The association with the biological process (BP) included keratinization, keratinocyte differentiation, and skin development; cellular components (CC) included cornified envelope, intermediate filament, and immunoglobulin complex; molecular function (MF) included structural constituent of epidermis, antigen binding, and peptidase inhibitor activity. KEGG included Ras signaling pathway, salivary secretion, and vascular smooth muscle contraction (Table 1).

**Table 1:** GO/KEGG functional enrichment of TIM-related DEGs.

Ontology	ID	Description	*p*.adj	qvalue	zscore
BP	GO:0031424	keratinization	8.89 × 10^−53^	8.45 × 10^−53^	−7.15
BP	GO:0030216	keratinocyte differentiation	4.57 × 10^−52^	4.34 × 10^−52^	−7.49
BP	GO:0043588	skin development	2.58 × 10^−51^	2.45 × 10^−51^	−7.88
BP	GO:0009913	epidermal cell differentiation	1.17 × 10^−50^	1.11 × 10^−50^	−7.62
BP	GO:0008544	epidermis development	1.50 × 10^−48^	1.43 × 10^−48^	−7.88
BP	GO:0070268	cornification	3.57 × 10^−42^	3.39 × 10^−42^	−5.84
CC	GO:0001533	cornified envelope	9.52 × 10^−38^	9.13 × 10^−38^	−5.48
CC	GO:0005882	intermediate filament	5.05 × 10^−13^	4.84 × 10^−13^	−4.38
CC	GO:0019814	immunoglobulin complex	1.06 × 10^−12^	1.01 × 10^−12^	−4.47
CC	GO:0045095	keratin filament	3.50 × 10^−12^	3.35 × 10^−12^	−3.50
CC	GO:0045111	intermediate filament cytoskeleton	6.31 × 10^−12^	6.05 × 10^−12^	−4.38
CC	GO:0072562	blood microparticle	9.07 × 10^−5^	8.70 × 10^−5^	−2.71
MF	GO:0030280	structural constituent of epidermis	1.54 × 10^−7^	1.43 × 10^−7^	−2.65
MF	GO:0003823	antigen binding	1.39 × 10^−4^	1.29 × 10^−4^	−3.46
MF	GO:0004866	endopeptidase inhibitor activity	0.004	0.004	−3.16
MF	GO:0030414	peptidase inhibitor activity	0.004	0.004	−3.16
MF	GO:0061135	endopeptidase regulator activity	0.004	0.004	−3.16
MF	GO:0004857	enzyme inhibitor activity	0.055	0.051	−3.46
KEGG	hsa05150	Staphylococcus aureus infection	2.80 × 10^−4^	2.28 × 10^−4^	−2.82
KEGG	hsa04915	Estrogen signaling pathway	2.80 × 10^−4^	2.28 × 10^−4^	−3.00
KEGG	hsa00590	Arachidonic acid metabolism	9.82 × 10^−4^	7.98 × 10^−4^	−2.45
KEGG	hsa04970	Salivary secretion	0.004	0.004	−1.63
KEGG	hsa04270	Vascular smooth muscle contraction	0.022	0.017	−2.45
KEGG	hsa04014	Ras signaling pathway	0.059	0.048	−2.65

**Figure 3:**
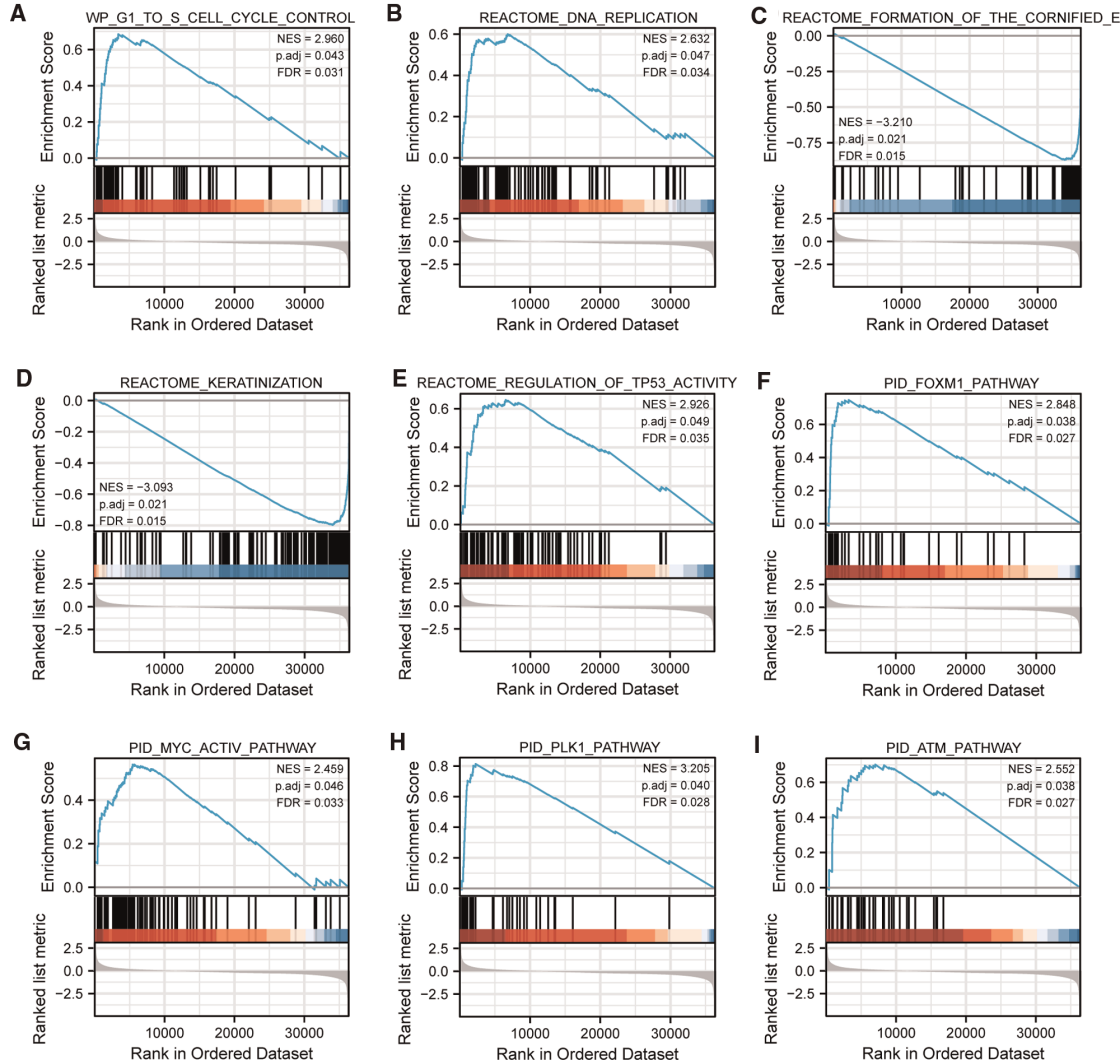
Representative GSEA enrichment plots of DEGs between high- and low-TIM expression groups. (**A–I**) NES, normalized enrichment score; *p*.adj, adjusted *p*-value; FDR, false discovery rate.

To gain further insight into the biologic pathways involved in SKCM with different TIM expression levels, GSEA was performed to identify critical signaling pathways, which showed DEGs significantly enriched in cell proliferation related clusters (Figures 3A,B), including cell cycle control, and DNA replication. Besides, TIM-related DEGs were also associated with formation of cornified envelope (Figure 3C), and keratinization (Figure 3D). More importantly, DEGs were also enriched in cancer pathways (Figures 3E–I), such as TP53 activity, FOXM1 pathway, MYC activity, PLK1 pathway, and ATM pathway. These pathways (Supplementary Table S2) were observed in the enrichment of MSigDB Collection (C2.all.v7.0.symbols.gmt) and the criterion for significant difference was set at FDR < 0.05, *p.*adj < 0.05.

### Association of TIM and Immune Cell Infiltration in SKCM

**Figure 4:**
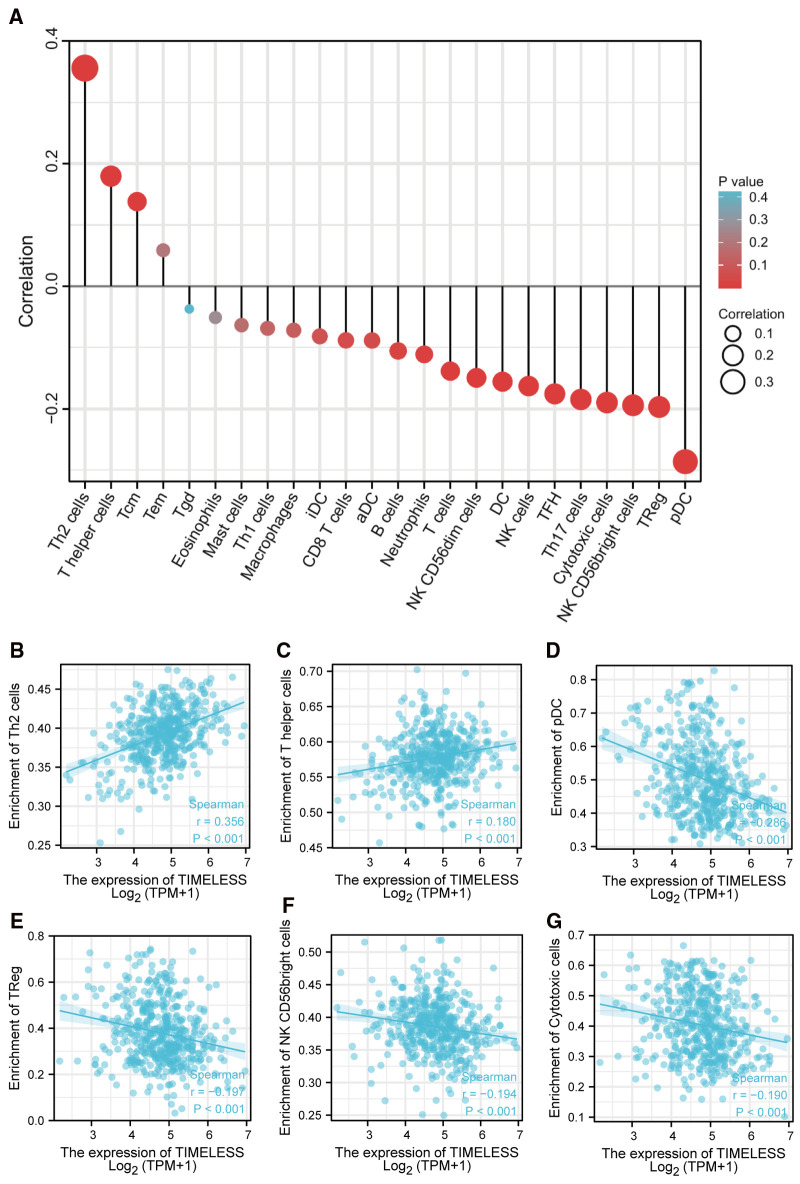
The expression of TIM was associated with immune infiltration in the SKCM microenvironment. (**A**) Relationships among infiltration levels of 24 immune cell types and TIM expression profiles by Spearmans analysis. The comparison of infiltration levels of most correlated immune cells, including (**B**) Th2 cells, (**C**) T helper cells, (**D**) pDC, (**E**) TReg, (**F**) NK CD56bright cells, and (**G**) cytotoxic cells was shown.

Spearman correlation analysis showed that the expression level of TIM in the SKCM microenvironment was correlated with the immune cell infiltration level quantified by ssGSEA. As shown in Figure 4A, Th2 cells, and T helper cells, were both positively correlated with TIM expression. However, pDC, TReg, NK CD56bright cells, and cytotoxic cells showed a negative association with TIM. More specifically, we evaluated the infiltration levels of six most relevant immune cells—Th2 cells (Figure 4B, *R* = 0.356, *p* < 0.001), T helper cells (Figure 4C, *R* = 0.180, *p* < 0.001), pDC (Figure 4D, *R* = −0.286, *p *< 0.001), TReg (Figure 4E, *R* = −0.197, *p* < 0.001), NK CD56bright (Figure 4F, *R* = −0.194, *p* < 0.001), and cytotoxic cells (Figure 4G, *R* = −0.190, *p* < 0.001)—in distinct TIM subgroups, which showed results consistent with those in Figure 4A.

### Association of Clinicopathological Characteristics and TIM Expression in SKCM Patients

We examined the clinicopathological features of SKCM patients in the two distinct TIM expression subgroups. The main clinicopathological characteristics of SKCM patients were presented in Table 2. There was no significant difference in gender, age, body mass index (BMI), TNM stages, radiation therapy, ulceration, and Breslow depth between the two groups. However, there was a significant difference between the two subgroups in the composition of N stage, melanoma ulceration, melanoma Clark level, overall survival (OS) event, and disease specific survival (DSS) event.

**Table 2:** Association between TIM expression and clinicopathologic features in SKCM patients from the TCGA database.

Characteristic	levels	TIM expression		*p*	statistic
		Low	High		
T stage, n	T1/T2/T3/T4	16/38/44/85	25/41/47/68	0.254	*x*^2 ^= 4.07
N stage, n	N0/N1/N2/N3	100/50/25/25	135/24/24/31	**0**.**002**	*x*^2 ^= 14.55
M stage, n	M0/M1	205/11	213/14	0.776	*x*^2 ^= 0.08
Pathologic stage, n	Stage I/II/III/IV	31/68/98/11	46/72/73/13	0.078	*x*^2 ^= 6.82
Radiation therapy, n	No/Yes	196/34	187/47	0.167	*x*^2 ^= 1.91
Gender, n	Female/Male	95/140	84/152	0.324	*x*^2 ^= 0.97
Age, n	≤60/>60	134/97	118/114	0.147	*x*^2 ^= 2.10
BMI, n	≤25/>25	45/82	39/85	0.593	*x*^2 ^= 0.29
Melanoma ulceration, n	No/Yes	60/91	87/76	**0**.**021**	*x*^2 ^= 5.32
Melanoma Clark level, n	I/II/III/IV/V	3/4/30/93/29	3/14/47/75/24	**0**.**020**	*x*^2 ^= 11.66
Breslow depth, n	≤3/>3	87/93	98/82	0.292	*x*^2 ^= 1.11
OS event, n (%)	Alive/Dead	142/91	105/126	**0**.**001**	*x*^2 ^= 10.57
DSS event, n (%)	Alive/Dead	152/79	115/112	**0**.**001**	*x*^2 ^= 10.18

*All bold values represent P values less than 0.05 to highlight statistical differences.*.

### Predictive Value of TIM for SKCM Diagnosis and Prognosis

**Figure 5:**
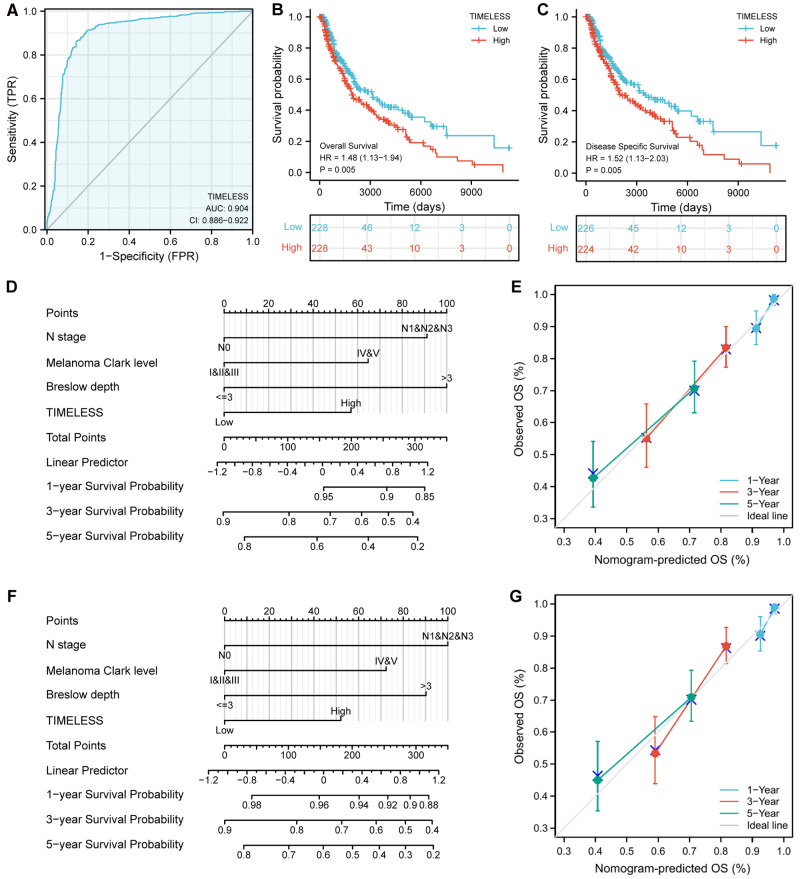
Predictive value of TIM for diagnosis and prognosis in SKCM patients. (**A**) ROC curve analysis evaluating the performance of TIM for SKCM diagnosis. Shown were the K-M analyses comparing OS (**B**), and DSS (**C**) between the two distinct TIM expression subgroups. (**D–G**) Construction and validation of nomograms based on TIM expression. Shown were the nomograms constructed to predict the probability of 1-, 3-, 5-year OS (**D**), and DSS (**F**) for SKCM. Calibration plots of OS, and DSS were shown in (**E,G**).

Univariate and multivariate Cox proportional hazards analysis was performed to examine the clinical advantages of TIM. As shown in Tables 3, 4, the expression level of TIM is an independent risk factor for OS (HR = 1.724, *p* = 0.009) and DSS (HR = 1.593, *p* = 0.034). Subsequently, we used ROC curve to demonstrate its value on discriminating SKCM diagnosis. As the area under the curve (AUC) was 0.904, TIM showed significant high sensitivity and specificity for SKCM diagnosis (Figure 5A). Subsequently, K-M analyses were applied to verify the prediction of TIM on clinical outcomes. As shown in Figures 5B, C, OS (HR = 1.48, *p *= 0.005), and DSS (HR = 1.52, *p *= 0.005) in the high-TIM expression group were both statistically worse than those in the low-TIM expression group.

**Table 3:** Univariate and multivariate Cox proportional hazards analysis of TIM expression and OS for SKCM patients.

Characteristics	Univariate analysis		Multivariate analysis	
	Hazard ratio (95% CI)	*p* value	Hazard ratio (95% CI)	*p* value
T stage (T1&T2 vs. T3&T4)	2.085 (1.501–2.895)	**<0** **.** **001**	0.890 (0.506–1.567)	0.687
N stage (N0 vs. N1&N2&N3)	1.752 (1.304–2.354)	**<0**.**001**	4.844 (1.425–16.464)	**0**.**011**
M stage (M0 vs. M1)	1.897 (1.029–3.496)	**0**.**040**	2.469 (0.935–6.520)	0.068
Age (≤60 vs.>60)	1.656 (1.251–2.192)	**<0**.**001**	0.949 (0.625–1.440)	0.806
Gender (Female vs. Male)	1.172 (0.879–1.563)	0.281		
Pathologic stage (I&II vs. III&IV)	1.617 (1.207–2.165)	**0**.**001**	0.436 (0.127–1.503)	0.189
Melanoma ulceration (No vs. Yes)	2.085 (1.495–2.907)	**<0**.**001**	1.521 (0.998–2.316)	0.051
Melanoma Clark level (I&II&III vs. IV&V)	2.167 (1.508–3.113)	**<0**.**001**	1.793 (1.077–2.986)	**0**.**025**
BMI (≤25 vs. >25)	0.827 (0.513–1.333)	0.436		
Breslow depth (≤3 vs. >3)	2.651 (1.938–3.627)	**<0**.**001**	1.981 (1.169–3.357)	**0**.**011**
Radiation therapy (No vs. Yes)	0.977 (0.694–1.377)	0.895		
TIM (Low vs. High)	1.480 (1.128–1.942)	**0**.**005**	1.724 (1.144–2.600)	**0**.**009**

*All bold values represent P values less than 0.05 to highlight statistical differences*.

**Table 4:** Univariate and multivariate Cox proportional hazards analysis of TIM expression and DSS for SKCM patients

Characteristics	Univariate analysis		Multivariate analysis	
	Hazard ratio (95% CI)	*p* value	Hazard ratio (95% CI)	*p* value
T stage (T1&T2 vs. T3&T4)	1.887 (1.341–2.654)	**<0** **.** **001**	0.925 (0.520–1.645)	0.791
N stage (N0 vs. N1&N2&N3)	1.665 (1.214–2.283)	**0**.**002**	6.914 (1.586–30.145)	**0**.**010**
M stage (M0 vs. M1)	2.200 (1.190–4.069)	**0**.**012**	2.758 (1.035–7.350)	**0**.**043**
Age (≤60 vs.>60)	1.699 (1.258–2.294)	**<0**.**001**	0.952 (0.613–1.480)	0.827
Gender (Female vs. Male)	1.161 (0.855–1.575)	0.340		
Pathologic stage (I&II vs. III&IV)	1.536 (1.125–2.096)	**0**.**007**	0.306 (0.069–1.356)	0.119
Melanoma ulceration (No vs. Yes)	1.948 (1.372–2.767)	**<0**.**001**	1.466 (0.944–2.277)	0.088
Melanoma Clark level (I&II&III vs. IV&V)	2.128 (1.457–3.108)	**<0**.**001**	1.813 (1.063–3.093)	**0**.**029**
BMI (≤25 vs. >25)	0.937 (0.545–1.612)	0.815		
Breslow depth (≤3 vs. >3)	2.274 (1.628–3.177)	**<0**.**001**	1.749 (1.019–3.001)	**0**.**042**
Radiation therapy (No vs. Yes)	0.994 (0.689–1.433)	0.973		
TIM (Low vs. High)	1.517 (1.134–2.029)	**0**.**005**	1.593 (1.035–2.452)	**0**.**034**

*All bold values represent P values less than 0.05 to highlight statistical differences*.

Moreover, to better predict the prognosis of SKCM patients, we constructed nomograms based on the results of multivariate Cox regression analysis. Four prognostic factors with statistical differences were included in the model, including clinical N stages, melanoma Clark level, Breslow depth, and TIM expression. As shown in Figures 5D,F, nomograms predicted OS and DSS at 1, 3, and 5 years, in which high expression of TIM predicted worse OS and DSS than low expression. Additionally, we also constructed calibration plots to evaluate the prediction accuracy of two nomogram models. As shown in Figures 5E, G, the predicted results were highly consistent with actual survival outcomes, with the exception of the 5-year prediction for OS and DSS, which was slightly overestimated.

### Promoter Methylation and Genetic Alterations of TIM in SKCM

**Figure 6:**
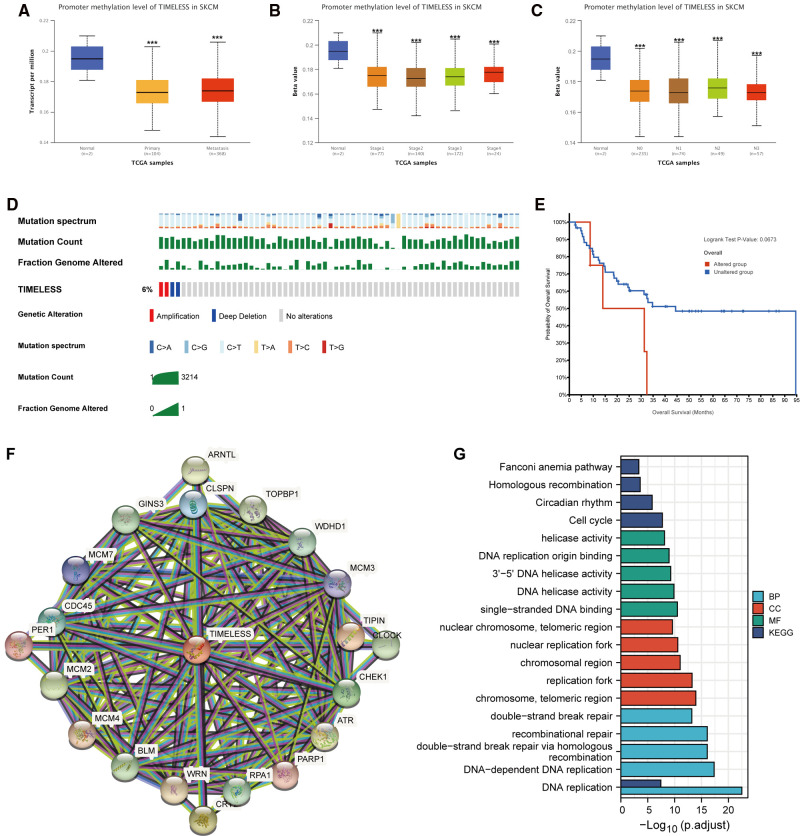
Promoter methylation and genetic alterations of TIM and potential regulatory mechanism of TIM in regulating SKCM. (**A–C**) Relationship between tumor type, stage, and nodal metastasis status of SKCM with the promoter methylation of TIM determined using UALCAN. (**D**) Genetic alterations of TIM in SKCM patients determined using cBioportal. (**E**) OS for TIM alterations was analyzed using cBioportal. (**F**) The neighbor gene network of 20 genes was constructed using STRING. (**G**) The bar diagrams showed the top five terms for each GO category as well as for the KEGG pathway analysis. ***p < 0.001.

To explore how TIM affects the prognosis of SKCM, we first explored the methylation levels and genetic mutations of TIM. Through the UALCAN database, we found that promoter methylation of TIM was lower in SKCM patients with both primary or metastatic tumors than in the normal group (Figure 6A). Moreover, the tumor stage and nodal metastasis status of SKCM also supported this conclusion (Figures 6B, C). Meanwhile, as shown in Figure 6D, TIM was altered in 6% of the queried SKCM patients. According to K-M analysis, patients with TIM alterations demonstrated shorter OS (Figure 6E).

Next, to further investigate the mechanisms behind TIM function, PPI network and GO/KEGG enrichment analysis was performed using STRING. Both PPI network and GO/KEGG pathways showed that TIM was related to DNA replication-related genes, including TOPBP1, WDHD1, TIPIN, CLSPN, and GINS3, and the cell cycle pathway (Figures 6F,G). These results indicated that TIM may regulate SKCM via regulating DNA replication and cell cycle.

### IHC Validation of TIM Expression

**Figure 7:**
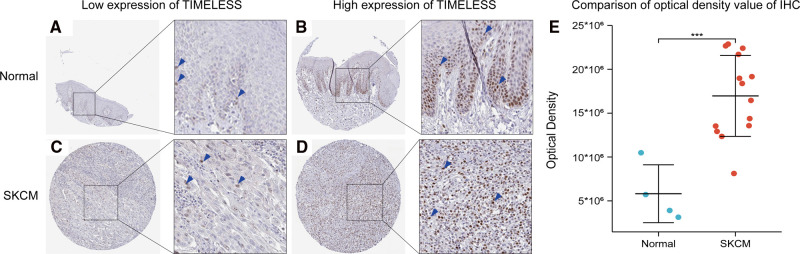
Representative IHC staining images of TIM in SKCM tissues and normal skin controls. (**A,B**) TIM was low (**A**) or high (**B**) in normal skin tissues. (**C,D**) TIM was low (**A**) or high (**B**) in SKCM tissues. (**E**) comparison of optical density value of IHC between SKCM tissues and normal skin controls. Blue arrows indicate positive staining.

To verify the former bioinformatic analysis results, we compared the gene expression of TIM in SKCM tissues vs normal skin controls using IHC data from the Human Protein Atlas (HPA) database. Representative images and analysis results were presented in Figure 7, the OD value of SKCM group was significantly higher than that of normal skin group (*p* < 0.05), consistent with the previous consequences.

## Discussion

In the present study, our results disclosed for the first time that TIM was overexpressed in SKCM tissues relative to normal tissues, which was also associated with poor prognosis. In addition, TIM-specific DEGs, primarily the up-regulated ones, were enriched in cell cycle, and several oncogenic signaling-related pathways. On the contrary, down-regulated DEGs were specifically enriched in keratinization. Subsequently, we assessed the relationship between the expression of TIM and immune infiltration. Besides, promoter methylation and genetic alterations of TIM in regulating SKCM were also further elucidated. Finally, we uncovered the value of TIM in the diagnosis of SKCM and revealed that higher expression of TIM was associated with worse clinical outcomes, such as OS and DSS.

The genes controlling accurate DNA replication were essential for normal cell proliferation, as replication errors could lead to abnormal cell proliferation. TIM was a well-characterized DNA replication regulator, and emerging evidence suggested that it played an important role in various cancers (4). In this study, we found that TIM expression was significantly elevated in a variety of solid cancers, including esophageal carcinoma, head and neck squamous cell carcinoma, and lung squamous cell carcinoma. Furthermore, TIM was also upregulated in other tumor types, such as bladder urothelial carcinoma, breast invasive carcinoma, colon adenocarcinoma, brain lower grade glioma, rectum adenocarcinoma, and uterine carcinosarcoma. Therefore, TIM may act as a key hub gene in tumor genesis and development.

We then sought to identify the biological functions and mechanisms involving TIM in SKCM. TIM, as a classic molecular clockwork in mammals, has been shown not only to be a circadian regulator, but essential for early embryonic development, DNA replication, and DNA damage repair (21–25). In this study, based on the functional annotation of TIM-associated DEGs, epidermal cell differentiation, keratinocyte differentiation, and skin development were closely related to TIM expression. In addition, according to the GSEA results, several cell cycle-related events and oncogenic related pathways were enriched in the high-TIM subgroup, while the downregulated DEGs were enriched in keratinization, and formation of the cornified envelope. The above results indicated that TIM was not only a key regulator of DNA replication in SKCM, but also an inhibitor of keratinization. Several studies have confirmed that TIM knockdown suppressed cancer cell proliferation and clonogenic growth in colorectal cancer, lung cancer, breast and cervical cancer (8, 9, 26). Not only that, studies have verified that each melanocyte contacts with about 30 keratinocytes in the basal and suprabasal layers forming the epidermal-melanin unit (27, 28). The gap junctional intercellular communication (GJIC) formed by these two types of cells was essential for cell homeostasis. The inability to establish melanocyte-keratinocyte GJIC may contribute to the development of SKCM (29). Our functional enrichment and GSEA results confirmed that TIM inhibited keratinization while promoting cell cycle, which may be one of the reasons for the poor prognosis of SKCM. More importantly, almost all tumorigenic-related pathways enriched in the high-TIM subgroup, including TP53, FOXM1, MYC, PLK1, and ATM signaling pathways, were related to regulating cell cycle, strongly suggesting the role of TIM to accelerate DNA replication and promote tumor cell proliferation, thereby worsening the prognosis of SKCM (30–33). Therefore, TIM expression might have important significance in SKCM tumorigenesis by regulating cell cycle and inhibiting keratinization.

In immune cell infiltration analysis, TIM expression was negatively correlated with pDC, TReg, NK CD56bright cells, Cytotoxic cells, and Th17 cells. Plasmacytoid dendritic cells (pDCs) is a subset of DCs denoted by their capacity to yield extensive amount of type I interferon (IFN-α) (34). In addition to IFN-α production, pDCs can also serve as antigen-presenting cells (APCs) and regulate immune responses to various antigens. The significant role played by pDCs in regulating both the innate and adaptive components of the immune system makes them a critical player in cancer immunology. Cancers that can be detected clinically must have escaped anti-tumor immune responses to grow progressively (35). Therefore, pDCs, negatively related to TIM expression, may contribute to the immune escape of tumor cells in SKCM. In the subsequent immune response, NK cells and cytotoxic cells can cooperate to kill the tumor cells (36). Unfortunately, over-expression of TIM also caused a decrease in infiltrating cytotoxic cells and NK cells. On the other hand, we found a significant positive correlation between TIM expression and Th2 infiltrating. Although Th2 cells were best known for anti-parasites and allergic reactions, their regulation and function in the tumor microenvironment (TME) were highly controversial (37). However, emerging evidence showed that Th2 cells promoted cancer genesis, progression, and metastasis. Some studies showed that Th2 cells in the TME were related to the progression of breast cancer and cervical neoplasia (38, 39). Besides, Th2 cells had been shown to promote metastasis in breast cancer, colorectal cancer, and lung cancer (40–43). Hence, increased Th2 cell infiltration in the TME caused by TIM expression might have a role in SKCM tumorigenesis.

Another important issue was the clinical significance of TIM in SKCM. AUC of the ROC curve for TIM discrimination in SKCM diagnosis was 0.904, strongly suggesting that TIM was a reliable biomarker for SKCM diagnosis. Moreover, K-M survival analyses showed that patients with high TIM expression had a strikingly worse OS and DSS than patients with low TIM expression. Furthermore, the Cox analyses of this study demonstrated that TIM might be an independent predictive factor of poor prognosis for SKCM, in which N stage, melanoma Clark level, Breslow depth, and TIM expression were independent prognostic factors for OS and DSS deterioration. In addition, not only was the prognostic value of TIM confirmed in SKCM, but its expression level in a variety of tumors was negatively correlated with OS, such as papillary renal cell carcinoma (44), adrenocortical carcinoma (45), breast invasive carcinoma (46), and lung cancer (47). From the above results, it could be seen that TIM was highly expressed in these tumors and was often associated with poor prognosis, which further confirmed that it was a common prognostic biomarker for multiple tumor types.

As for TIM gene alternative and DNA methylation, results of Fu et al. demonstrated that hypomethylation of TIM was associated with poorer prognosis and advanced stages of breast cancer (6). These results were consistent with later observations that higher TIM was related to shorter metastatic survival, especially in ER-positive/HER2-negative women (48). According to these results, it can be hypothesized that TIM overexpression might be the result of hypomethylation in the promoter region. In our study, analysis of UALCAN revealed that TIM gene promoter methylation was lower in both primary and metastatic SKCM compared to normal controls. Few studies have scrutinized the role of TIM mutations in SKCM. Among the surveyed SKCM patients, 6% had TIM mutations, which indicated a shorter OS. These findings suggested the promoter methylation and genetic alterations of TIM played an essential role in SKCM prognosis. Therefore, further research is needed in order to fully clarify the role of promoter methylation and genetic alterations in SKCM patients.

In order to further understand the biological functions of TIM, we constructed a PPI network using STRING database and conducted functional enrichment analysis on these genes. The results showed that the functions of these genes were mainly enriched in DNA replication and cell cycle, further supporting the above predicted results. Increased expression of TIM may affect the tumogenesis and prognosis of SKCM by promoting the proliferation of melanoma cells.

The results of IHC indicated that the expression of TIM in SKCM tissues was higher than that in normal skin tissues, which was consistent with the previous prediction. Although we have disclosed the potential mechanism of TIM in SKCM tumorigenesis and its prognostic value in SKCM clinical outcomes, several factors could limit the extent to which the results can be generalized. First, as a potential novel therapeutic target, the sample size should be expanded in further research to enhance the reliability and representativeness of the results. Second, since we mainly focused on RNA-seq data from TCGA and GTEx databases, the results could be further verified by in vivo and in vitro research.

In conclusion, the highly expressed TIM in SKCM may play a pivotal role in tumorigenesis by regulating cell cycle and inhibiting melanocyte-keratinocyte GJIC. In addition, it also has prognostic value for clinical outcomes. Our results shed light on TIM as a potential therapeutic target for SKCM.

## Data Availability

The datasets presented in this study can be found in online repositories. The names of the repository/repositories and accession number(s) can be found in the article/Supplementary Material.
